# Physical and Social Neighborhood Environments and Well-Being

**DOI:** 10.1093/geroni/igaa057.1421

**Published:** 2020-12-16

**Authors:** Midori Takayama, Yoshiko Ishioka, Ikuko Sugawara

**Affiliations:** 1 Keio University, Yokohama, Japan; 2 Keio University, Minato-ku, Kanagawa, Japan; 3 University of Tokyo, Tokyo, Tokyo, Japan

## Abstract

Existing research has found effects of neighborhood environment on well-being. However, it is still not clear what features of neighborhood environments affect well-being for older adults and whether the impact of the environment varies depending on the health and economic conditions. In this study, we examined the relations between 4 physical and social neighborhood-context factors, that is, the availability of neighborhood physical resources (e.g. community centers and libraries), the walkability and accessibility, the availability of social resources (e.g. culture and recreation programs, and social care services), and the social inclusion (e.g. participation in decision making, and positive social attitude toward older adults), and individual-level well-being. Moreover, we examined the health and economic disparities of effect of neighborhood environments on well-being. We used data from locally representative longitudinal study of older Japanese aged 74 to 86 (N = 1388). Results from multi-level linear regression showed that after controlling individual variables having inhibitory/facilitatory effects of well-being, the availability of physical resources was associated with higher well-being score. Especially among older adults who had financial strains, the availability of physical environment had a positive effect on well-being more strongly. For older adults who had better physical functions, accessibility and walkability were associated with higher well-being score. The social inclusiveness was associated with higher well-being score among those who had no financial strain. These important findings demonstrate the need for more research exploring the underlying mechanisms. The potential benefits of this approach provide a basis for developing models of maintaining well-being for older adults.

